# Health-related quality of life and associated factors in functionally independent older people

**DOI:** 10.1186/s12877-016-0410-3

**Published:** 2017-01-14

**Authors:** Mónica Machón, Isabel Larrañaga, Miren Dorronsoro, Kalliopi Vrotsou, Itziar Vergara

**Affiliations:** 1 Unidad de Investigación de Atención Primaria-OSIS Gipuzkoa, Osakidetza, Instituto Biodonostia, Paseo Doctor Beguiristian s/n, San Sebastián, 20014 Spain; 2 Red de Investigación en Servicios de Salud en Enfermedades Crónicas (REDISSEC), Bilbao, Spain; 3 Instituto de Investigación Sanitaria Biodonostia, San Sebastián, Spain; 4 Departamento de Salud, Delegación Territorial de Gipuzkoa, Gobierno Vasco, San Sebastián, Spain; 5 Dirección de Salud Pública y Adicciones, Gobierno Vasco, Vitoria, Spain; 6 CIBER Epidemiología y Salud Pública (CIBERESP), Madrid, Spain

**Keywords:** Health-related quality of life, Older people, Living conditions, EuroQol-5D

## Abstract

**Background:**

Health-related quality of life (HRQL) is a key indicator of elderly people’s health status that can be affected by different factors. However, little is known about which variables are associated with it in functionally independent elderly people. The aim of this project was to study HRQL and a wide variety of health, lifestyle, social and contextual aspects and their relation to HRQL in a sample of functionally independent, non-cognitively impaired community-dwelling adults, over 65 years of age, from a northern region of Spain.

**Methods:**

A cross-sectional study for which data was collected by face-to-face interviews with the selected individuals. HRQL was measured with the EuroQol-5D scale, consisting of a 5 item descriptive system and a visual analogue scale (VAS). VAS values lower than 70 were considered poor HRQL. Binary logistic regression was used to identify factors related to the outcome.

**Results:**

Six hundred and thirty-four individuals were included in the study. The mean age was 74.8 (SD 6.7) years, 55% of the participants were women and 46% rated their HRQL as poor. Several variables were found to be significantly associated with a poor HRQL in the multivariate model, adjusted for age and sex: polypharmacy (OR: 2.32, 95% CI: 1.62–3.31), the presence of sensory impairment (OR: 1.83, 95% CI: 1.24–2.69), not being engaged in cognitively stimulating activities (OR: 2.51, 95% CI: 1.03–6.16), or in group social activities (OR: 1.57, 95% CI: 1.11–2.22), low level of social support (OR: 3.12, 95%CI: 1.78–5.46) and the presence of obstacles in the closest home environment (OR: 1.83, 95%CI: 1.11–3.02).

**Conclusions:**

The study identified a set of health, social and contextual variables as strongly related to HRQL in functionally independent community-dwelling older people. The results highlight the multidimensional nature of HRQL. They also reveal the importance of a comprehensive assessment of HRQL when designing adequate health-related programmes aiming to enhance active and healthy ageing and delay the onset of dependence.

## Background

As the populations around the world are rapidly ageing [1], health authorities [2] have a growing interest in preserving and promoting a healthy and active living and in maintaining the highest quality of life. In Spain, the population of people over the age of 65 has increased considerably, from 3.4 million in 1975 (10% of the total population) to 8.2 million (18% of the total population) in 2015 [3].

Health-related quality of life (HRQL) is an indicator of a person’s overall health status and it can be used in different contexts, such as clinical studies, health care economic evaluations or population health surveys [4]. A number of different conceptualizations of HRQL exist. Among the most referenced ones is that given by Patrick and Erikson [5], who defined HRQL as “The value assigned to the duration of life as modified by the impairments, functional states, perceptions and social opportunities that are influenced by disease, injury, treatment or policy”. Several models addressing this multidimensional concept have been proposed over the years [6–8], among which the Wilson and Cleary [9] is the most commonly used [8]. This model includes five domains, namely, biological variables, symptom status, functional status, general health perceptions, and overall quality of life; while it also incorporates individual and environmental characteristics. Each of the five domains has a direct effect on the successive domain (that is, biological variables influences symptom status and this influences functional status, and so on), while environmental and individual factors affect all domains, except for the biological variables. Ferrans et al. [6] offered a modified version of the previous model, by considering that environmental and individual characteristics actually affect all five domains [6]. HRQL, as a multifaceted and complex concept, can thus be influenced by several factors, such as sociodemographic characteristics, chronic diseases, functional status, social network or neighbourhood environment [10–14]. HRQL can be measured using either generic or specific questionnaires. The first are applicable to virtually any adult population and the latter to specific population groups [15]. Among the generic instruments, the SF-36, EuroQol- 5D and Nottingham Health Profile have good evidence as far as the psychometric properties of reliability, validity and responsiveness are concerned [15, 16].

When studied simultaneously, the effect of functional capacity usually shadows those of sociodemographic variables, chronic diseases, social characteristics or neighbourhood environment data. As a result, less is known about the actual role of the latter factors pertaining to three domains and two characteristics (biological variables, symptom status, general health perception and individual and environmental characteristics) of the Wilson and Cleary model, and their association with HRQL. This approach is relevant when wishing to design and implement strategies for promoting an active and healthy ageing and postponing the onset of dependence.

With this in mind, this study has two objectives: to describe HRQL and to examine the role of health, lifestyle, social and contextual factors pertaining to the Wilson and Cleary model, considering all of them in a comprehensive manner. To this end, a sample of community dwelling and functionally independent older people without cognitive deterioration was studied. Our hypothesis was that HRQL in functionally independent older adults is related to the previously described factors, and that the role of these factors varies when functional status is not considered. The current data are part of a bigger study aimed at understanding different aspects of health and living conditions of older people [17].

## Methods

### Study design and sample size

This was a cross-sectional study conducted in the province of Gipuzkoa (Spain), with an estimated population of 708,000 inhabitants [18]. A random sample of 800 individuals over 65 years of age was selected based on a multi-stage sampling approach. First, a sample of 15 municipalities, of the 88 existing in the region, was randomly selected. Different municipality sizes, <2000, 2001–10000, 10001–50000 and >50000 people, were meant to be equally represented in this sample, providing each 25% of the latter. A total of 7, 4, 3 and 1 municipalities were selected for each size, respectively. The age and sex distribution of the sample was set to be the same as that of the local population of interest [19]. Participants were captured until completion of the estimated sample size of 800 individuals was fulfilled. Afterwards, and for the purpose of this work, participants with cognitive impairment, determined by the Pfeiffer’s Short Portable Mental Status Questionnaire (that is, three or more errors for those who could write and read and four or more errors for illiterate subjects) [20, 21], were excluded from the study as well as participants with dependence, defined as having less than 95 points in the Barthel score [22, 23].

### Data collection

The fieldwork was conducted between January 2013 and February 2013. All participants were interviewed at their homes by trained interviewers using a battery of 145 questions on several health and living conditions aspects. The included questions were based on the published literature and were set with the help of a multidisciplinary group of experts. No open reply questions were asked. Questions requiring a frequency response (for example, number of prescription drugs consumed, cigarettes smoked per week), were answered in a numerical form. The presence of a partner, family, friend or caregiver during the interview was allowed if so desired by the participants as they may have felt more confident.

### Measurements

The main outcome of interest was HRQL, measured using the EuroQol EQ-5D-3 L scale [24]. The EQ-5D-3 L is a generic health status instrument consisting of two parts: the descriptive system or self-classifier and a visual analogue scale (EQ-VAS) measuring overall self-rated health status. The descriptive system measures health in five dimensions: mobility, self-care, usual activities, pain/discomfort, and anxiety/depression. Each dimension is measured on three levels of severity: no problems, some/moderate problems, and severe/extreme problems. The EQ-VAS is a 20 cm, vertical, hash-marked visual analogue scale on which respondents are asked to rate their overall health between 0 (worst imaginable health state) and 100 (best imaginable health state). In this study, the cut-off point was the median VAS obtained in the studied sample. This conceptualization was previously used by other authors [10, 25].

Furthermore, several sociodemographic, health, lifestyle, social and contextual variables associated with HRQL in the literature were also studied. Sociodemographic characteristics included sex, age, level of education, monthly family income and living arrangements.

Regarding health, several variables were examined. Cognitive impairment was detected by using the Pfeiffer’s Short Portable Mental Status Questionnaire [20, 21]. Functional status was assessed with the Barthel Index [22, 23]. Participants were also asked about diagnosed chronic conditions (for example, diabetes, hypertension, cardiovascular disease). Information about prescription drugs taken daily during the previous two weeks was collected and the consumption of three or more was considered polypharmacy [26]. The Geriatric Depression Scale [27, 28] was also implemented. This scale is composed of 15 items (10 positive and 5 negatively worded) and a score of 5 or more points indicates the presence of depressive symptoms. Participants also reported the number of falls they had in the past 12 months. The capacity of the participants to relate and engage with the environment was explored asking them whether they had hearing, vision, speech or chewing difficulties. People having at least one such difficulty were considered sensory impaired, understanding that in this case, sensory refers to the aforementioned capacity to relate to others and not only to sensory organs integrity [29].

Lifestyle habits such as smoking status and physical activity were recorded. Sleep quality, that refers to whether the hours the participant slept allowed them to rest well enough, was assessed. Undernutrition risk was evaluated with a 9-item screening tool [30, 31]. Its score ranges from 0 to 26 points and scores of 7 or higher indicate a person at risk of being undernourished or actually undernourished, presenting a bad nutrition pattern.

Regarding social variables, leisure time activities were studied. The participants were asked how frequently they engaged in a series of activities in the last 12 months, grouped in four activity types: cognitively stimulating activities (reading, listening to the radio); active leisure activities (going for a walk, taking care of a pet, gardening); social leisure activities (spending time with friends, attending sport events, going to a dance club, going to the cinema/theatre or a concert, going to a bar or out to have lunch/dinner); and group social activities (going on holiday). For the assessment of the first three, subjects were considered to be carrying out a certain type of activity if they usually engaged in any of the activities of that group.

Social support is a multidimensional construct that can be measured with different tools. The evaluation of social isolation was performed with the 6-item Lubben Social Network Scale [32, 33]. This is a 30 point measure, which evaluates older people’s perception of social support from family and friends. A score lower than 12 points was considered high risk of social isolation [32]. The Duke-UNC Functional Social Support Questionnaire was used (DUFSS) [34–36] to measure social support. It contains 11 items and its score ranges from 11 to 55, with a score of ≤ 32 points indicating a low level of social support.

Social capital, was studied with a three-item scale included in the *European Social Survey* [37]. Each question ranged from a 0 (low trust) to 10 (high trust). A total score of ≤ 5 points was considered to reflect low social trust and values >5 high social trust. Self-perceived social life was measured with a single item and response categories were grouped as follows: satisfactory (very satisfactory/satisfactory) and unsatisfactory (unsatisfactory/very unsatisfactory).

Finally, regarding contextual variables, participants were asked about the presence of home facilities, like heating; the presence of physical barriers or obstacles that could hinder their mobility inside their home and in the closest home environment (for example, steps at the entrance of the building, heavy door, walkway) and to rate their overall house condition, eventually categorised as good (responses: good, very good and excellent) or poor (responses: poor and fair). The Checklist of Essential Features of Age-friendly Cities from WHO [38] was used as a guide to develop items about community and neighbourhood resources. In total, 21 items asked about the following features: outdoor spaces (pavements, green spaces, public toilets and streets), public buildings (elevators, toilets and ramps) and public transport (accessible vehicles, priority seats and drop-off spots). Each item’s answers were grouped into two categories: none/very few/few and some/many/very many. Community services were considered adequate for the participants if they answered some/many/very many to questions about green spaces, public toilets, accessible public transport and priority seats on public transport. An additional item asking whether they walked or used transport to travel to a number of facilities, like shopping facilities, was included.

### Statistical analysis

Continuous variables were described as means with standard deviations (SD), and categorical variables as frequencies with percentages (%). Chi-square or Fisher’s exact tests and Student’s *t*-test were used to compare categorical and continuous variables, respectively. Univariate and multivariate binary logistic regression models, adjusted for age and sex were performed. All variables with p-values <0.10 in the univariate stage were taken into account in the multivariate analysis phase. Both backward and forward regression models were fitted. The multivariate model results are presented as odd ratios (OR) with their respective 95% confidence intervals (95% CI) and p-values. P-values <0.05 were considered statistically significant. Collinearity was assessed with the correlation matrix of the estimated parameters, their eigenvalues and proportion of variation [39]. The Hosmer-Lemneshow test, R-square and area under the curve (AUC) are given for the final model. SAS version 9.3 software was used for all statistical analyses.

## Results

Of the initial sample of 800 individuals, 125 and 41 were excluded from the study due to cognitive impairment and BADL dependence, respectively. Compared to the included individuals, those excluded were mostly women (67%), were older (*p <* 0.0001) and had poorer health, with a higher percentage having ≥3 chronic conditions (*p <* 0.0001) and taking ≥3 prescription drugs (*p <* 0.0001). Excluded individuals did not modify in excess the age and sex distribution of the initial sample, as the final sample categories were between 1–5% of the latter. The study sample was comprised of 634 independent community-dwelling older adults with a mean age of 74.8 (SD 6.7) years, of which 55% were women. EQ-VAS scores ranged between 10 and 100 points with a median value of 70 points. Considering <70 scores to indicate poor health, 46% of the participants rated their HRQL as such. In 126 interviews an accompanying person was present and in 12 (2%) the participants answered the battery of questions with some help from that person. Table 1 describes the distribution of subjects according to EQ-5D dimensions and the mean EQ-VAS values. The dimensions more frequently reported to cause some/moderate problems were pain/discomfort (28%) followed by mobility (13%) and anxiety/depression (10%).

**Table 1:** Frequency of replies in the EuroQol 5D scale

EuroQol 5D scale	Total sample (*n =* 634)
EQ-5D dimensions, n (%)	
Mobility	
No problems	550 (87)
Moderate problems	84 (13)
Extreme problems	0
Self-care	
No	617 (97)
Moderate	17 (3)
Extreme	0
Usual activities	
No	582 (92)
Moderate	47 (7)
Extreme	5 (1)
Pain/discomfort	
No	446 (70)
Moderate	175 (28)
Extreme	13 (2)
Anxiety/depression	
No	558 (88)
Moderate	66 (10)
Extreme	10 (2)
EQ-VAS score	
Mean (SD)	67 (16)
Median (Q1-Q3)	70 (55-80)

Numbers are n (%) unless otherwise stated

*SD* standard deviation, *EQ*-*VAS* EuroQol visual analogue scale, Q1 and Q3 represent the first and third quartile respectively

### Univariate analysis

A significantly higher proportion of respondents with a secondary or higher educational levels (*p =* 0.028), and those with a monthly family income higher than 1500 euros (*p =* 0.002), reported that their HRQL was good compared to those with lower educational level and incomes (Table 2).

**Table 2:** Characteristics of older people according to their HRQL

Variables	Health-Related Quality of Life		
	Poor (*n =* 292)	Good (*n =* 342)	p-value
Sex			
Men	125 (44)	160 (56)	0.316
Women	167 (48)	182 (52)	
Age in years, mean (SD)	75.1 (6.6)	74.6 (6.7)	0.378
Level of education			
Primary or lower	250 (48)	269 (52)	0.028
Secondary or higher	42 (37)	72 (63)	
Missing	0	1	
Monthly family income (€)			
≤ 1,500 euros	178 (52)	167 (48)	0.002
≥ 1,501 euros	53 (37)	92 (63)	
Missing	61	83	
Living arrangements			
Alone	77 (48)	82 (52)	0.502
With others	215 (45)	259 (55)	
Missing	0	1	
Number of diagnosed chronic diseases			
0-2	176 (39)	272 (61)	<0.0001
≥ 3	116 (62)	70 (38)	
Number of drugs consumed daily			
0-2	164 (39)	258 (61)	<0.0001
≥ 3	128 (60)	84 (40)	
GDS score			
Not depressive symptoms (<5)	235 (42)	321 (58)	<0.0001
Depressive symptoms (≥5)	57 (73)	21 (27)	
Falls in the previous 12 months			
0	231 (45)	284 (55)	0.206
≥ 1	61 (51)	58 (49)	
Sensory impairment			
Yes	94 (58)	69 (42)	0.001
No	198 (42)	273 (58)	
Smoker			
Yes	19 (45)	23 (55)	0.921
No	272 (46)	319 (54)	
Missing	1	0	
Physical activity in the previous 2 weeks			
Yes	232 (44)	299 (56)	0.007
No	60 (58)	43 (42)	
Sleep quality			
Good	243 (44)	309 (56)	0.008
Bad	49 (60)	33 (40)	
Nutrition pattern (STARU score)			
Good (<7)	268 (45)	331 (55)	0.006
Bad (≥7)	24 (69)	11 (31)	
Cognitively stimulating activities			
Yes	274 (45)	334 (55)	0.016
No	18 (69)	8 (31)	
Active leisure activities			
Yes	282 (46)	331 (54)	0.884
No	10 (48)	11 (52)	
Social leisure activities			
Yes	252 (46)	300 (54)	0.596
No	40 (49)	42 (51)	
Group social activities			
Yes	144 (42)	202 (58)	0.014
No	147 (51)	139 (49)	
Missing	1	1	
LSNS score			
High risk of social isolation (<12)	76 (54)	64 (46)	0.027
Low risk of social isolation (≥12)	216 (44)	278 (56)	
Duke-UNC FSSQ score			
Low level of social support (≤32)	48 (67)	24 (33)	0.0002
High level of social support (>32)	244 (43)	318 (57)	
STS			
Low social trust (≤5)	124 (60)	84 (40)	<0.0001
High social trust (>5)	168 (39)	258 (61)	
Self-perceived social life			
*Uns*atisfactory	28 (65)	15 (35)	0.009
Satisfactory	264 (45)	327 (55)	
Heating			
Yes	222 (44)	285 (56)	0.022
No	70 (55)	57 (45)	
Obstacles inside their home			
Yes	35 (69)	16 (31)	0.001
No	256 (44)	324 (56)	
Missing	1	2	
Obstacles in the closest home environment			
Yes	57 (65)	31 (35)	0.0002
No	234 (43)	309 (57)	
Missing	1	2	
Self-perceived housing condition			
Good	265 (46)	313 (54)	0.734
Poor	27 (48)	29 (52)	
Adequate community services			
Adequate	251 (48)	273 (52)	0.042
Not adequate	41 (37)	69 (63)	
Shopping facilities within walking distance			
Yes	230 (45)	285 (55)	0.142
No	62 (52)	57 (48)	

Numbers are n (%) unless otherwise stated. Row percentages are presented. When there is missing data, frequencies do not add up to column totals

*GDS* Geriatric Depression Scale, *Duke*-*UNC FSSQ* Duke-UNC Functional Social Support Questionnaire, *STS* Social Trust Scale, *LSNS* Lubben Social Network Scale, *STARU* Screening Tool for Assessing Risk of Undernutrition

Regarding health variables, those presenting ≥3 chronic diseases and taking ≥3 drugs daily (*p <* 0.0001), having depressive symptoms (*p <* 0.0001) and a sensory impairment (*p =* 0.001) perceived their HRQL as worse than those without the above mentioned medical problems. Furthermore, individuals not engaged in any physical activity (*p =* 0.007), with bad sleep quality (*p =* 0.008) and a bad nutritional pattern (*p =* 0.006) were more likely to report a poor HRQL (Table 2).

Concerning social variables, the proportion of individuals perceiving their HRQL to be good was higher among those who engaged in some leisure activities, such as cognitively stimulating activities (*p =* 0.016) or group social activities (*p =* 0.014), with a low risk of social isolation (*p =* 0.027), a high level of social support (*p =* 0.0002) or social trust (*p <* 0.0001) and satisfactory self-perceived social life (*p =* 0.009), compared to those who did not engage in the social activities considered, had low social support or unsatisfactory social life.

Regarding contextual variables, those who had heating (*p =* 0.022), did not have obstacles inside their home (*p =* 0.001) and in the closest home environment (*p =* 0.0002) or perceived their community services as adequate (*p =* 0.042) were more likely to consider their HRQL good.

No statistically significant differences were observed for the following variables: age, sex, living arrangements, history of falls, smoking status, active or social leisure activities, housing condition and shopping facilities within walking distance (Table 2).

### Multivariate analysis

In the multivariate analysis stage, the variables that best explained HRQL, after adjusting for age and sex were: number of drugs consumed daily sensory-related abilities, engagement in leisure activities, social support and accessibility in the closest home environment (Table 3). The variables of daily consumed drugs and number of chronic diseases were strongly associated, meaning that only one of the two could be maintained in the multivariate model. Due to its ease of assessment, the number of drugs was finally preferred. Similarly, social support and social network variables were also correlated. We opted for the social support variable in our model, measured by the Duke scale, for considering it a more comprehensive tool.

**Table 3:** Factors associated with poor HRQL in older people

Variables	Adjusted OR (95% CI)	p-value
Age	0.99 (0.97-1.02)	0.530
Sex		
Women	1.00	0.552
Men	0.90 (0.64-1.27)	
Number of drugs consumed daily		
0-2	1.00	<0.0001
≥ 3	2.32 (1.62-3.31)	
Sensory impairment		
No	1.00	0.002
Yes	1.83 (1.24-2.69)	
Cognitively stimulating activities		
Yes	1.00	0.044
No	2.51 (1.03-6.16)	
Group social activities		
Yes	1.00	0.012
No	1.57 (1.11-2.22)	
DUFSS		
High level of social support (>32)	1.00	<0.0001
Low level of social support (≤32)	3.12 (1.78-5.46)	
Obstacles in the closest home environment		
No	1.00	0.019
Yes	1.83 (1.11-3.02)	

*STARU* Screening Tool for Assessing Risk of Undernutrition, *OR* Odds Ratio, 95% *CI* 95% confidence interval, DUFSS = Duke-UNC Questionnaire of Functional Social Support; OR > 1 indicates higher odds of poor HRQL; OR <1 indicates lower odds of poor HRQL; Estimates are based on *n =* 629 participants due to missing value; Model diagnostics Area Under the Curve = 0.700; Hosmer-Lemeshow-*p =* 0.169; R-square = 0.108; Max-Rescaled = 0.145

Individuals presenting polypharmacy were more likely to report poor HRQL (OR: 2.32, 95% CI: 1.62–3.31). Individuals with sensory impairment and those having obstacles in the closest home environment were around twice as likely to rate their HRQL as poor (OR: 1.83, 95%CI: 1.24–2.69; and OR: 1.83, 95% CI: 1.13–3.02). Similarly, individuals who did not engage in cognitively stimulating (OR: 2.51, 95% CI: 1.03–6.16) or group social activities (OR: 1.57, 95% CI: 1.11–2.22) and those with a low level of social support (OR: 3.12, 95% CI: 1.78–5.46) were more likely to evaluate their HRQL as poor, compared to those who engaged in these activities and those with high levels of social support. No collinearity was found among the variables included in the models. The AUC obtained for this model was 0.700, suggesting good discriminatory capacity [40].

## Discussion

The aim of this study was to examine the relationship between different health, lifestyle, social and contextual factors and HRQL in a group of functionally independent older people without cognitive impairment. Cognitively impaired subjects were excluded as their condition could compromise their ability to provide valid answers. A similar decision was taken in a few previous studies [10, 41]. The inclusion of subjects with dependence [10] affects the assessment of HRQL, as functional status has an impact on it. However, by excluding cognitively impaired and subjects presenting some level of dependence, this study moves a step forward in exploring HRQL. Disregarding functional status offers the opportunity to study other important factors and their association with HRQL. Thus, the main contribution of this study is to provide a more extensive knowledge on which factors beyond functionality are associated with HRQL in older people. This knowledge may be useful in the design of effective public health strategies for promoting an active and healthy lifestyle in this population group.

When exploring the Euroqol dimensions, moderate problems were more frequent in pain/discomfort and mobility. The proportions seen in our sample were much lower than the respective values obtained with elders, aged 75 or more, from six European countries (that is, 50% and 48%) [11]. These differences are reasonable given that we only included functionally independent subjects. Assessing the effects of all relevant factors in a multivariate model, adjusted for age and sex, those that best explained poor HRQL in our study were: polypharmacy, having sensory impairment, not engaging in certain leisure activities (cognitively stimulating activities or group social activities), low level of social support and the presence of obstacles in the closest home environment. These results confirm our hypothesis, except for the lifestyle variables, and are consistent with findings reported by other authors. In a Swedish sample of 350 subjects aged 85 increased medication use was associated to poor HRQL [42]. Furthermore, a Chinese study of community-dwelling people aged ≥65 years, found that visual and hearing abilities were significantly related to the physical and mental components summary scores of the SF-36 scale [41]. Social context was also an important factor in our study. Engaging in cognitively stimulating activities or group social activities and a high level of social support were associated with a better HRQL. In line with our results, a previous Spanish study on community-dwelling adults aged ≥60 found that social support, measured with DUFSS, had a positive effect on HRQL [13]. Similarly, in a German population-based cohort of 2443 people aged ≥75 years, a strong association was found between social support and HRQL (EQ-VAS) [43]. This may reflect the importance of maintaining good mental abilities and social relationships in older people as a way to enhance social connectedness and quality of life [44, 45].

In contrast to the findings of other published studies [14, 46], our data suggested no association between neighbourhood characteristics and HRQL, neither in the univariate (except for adequate community services) nor in the multivariate analysis. Several possible explanations are proposed. It is probable that subjects with poor HRQL go out less frequently and remain unaware of the actual situation of their community environments. It is also likely that people living in the same place for a long time get used to the environment and its particular characteristics. Another plausible explanation may be that the questions implemented to analyse this aspect are not suitable for use in older people or not valid to assess our neighbourhood characteristics. Finally, this may also be due to the design of the respective questions, which were taken from the WHO checklist for Essential Features of Age-friendly Cities [38] and are not part of a specific or validated scale. Further research will be done to explore this issue in more depth and perhaps, new instruments should be developed to examine the neighbourhood environment adequately in this population group.

It is important to note that housing variables were associated with HRQL in both univariate (presence of heating and obstacles inside their home and in the closest home environment) and multivariate analysis (presence of obstacles in the closest home environment). This is in agreement with the study of Windle et al. [47], who found that housing difficulties (including several items, like problems with steps/stairs) and being cold with current heating predicted poorer health status, measured with EQ-VAS scale, in a sample of 411 older people in Wales. Due to the fact that the home environment and its close surroundings become the main living spaces for older people [48], it is relevant to emphasize the importance of maintaining an appropriate living environment for delaying the onset of dependency and for enhancing quality of life.

The strengths of the study deserve to be described. Firstly, a relatively high number of community-dwelling functionally independent older people have been studied. In addition, a wide range of aspects related to health and living conditions, including the assessment of the neighbourhood environment, and their association with HRQL were explored. Another relevant characteristic of the study was the collaboration of a panel of multidisciplinary experts for selecting the battery of questions used during the fieldwork process.

In contrast, certain limitations should also be addressed. Since it was a cross-sectional study, no cause-effect relationships can be established. Nevertheless, this type of studies is very important for understanding which factors have an effect on health and for generating hypotheses for future research [49]. The extensive battery of questions required around 60 min interviews, fact that may be responsible for the participation of individuals with a relatively good functional status. Furthermore, it is possible that some variation may have been introduced in the data collection given that the data was gathered by 20 interviewers. Nonetheless, all interviewers were experienced in this fieldwork and were additionally instructed before the initiation of the current project which should have minimized any possible variations. Similar situations have been described in other studies [50]. It is well known that face-to-face interviews are the recommended method to collect information when using long questionnaires, and higher response rates are obtained compared to postal or telephone interviews [51]. As a consequence of the data collection method, missing data was not a particular problem in this study. The only exception was income level, left unanswered by 22% of the sample. It is possible that more information on this variable may have allowed for further explorations of this particular well-being aspect. Furthermore, information on chronic conditions was self-reported by the participants, a fact that may have affected the reported figures. Nevertheless, given the study design, time frame and the difficulty in accessing a patient’s medical history, this approach regularly seen in the literature [12, 13] was the most suitable one. The refusal rate and the characteristics of the subjects declined to participate were not registered during the data collection phase. There is a possibility that this may have affected the reproducibility of the presented results. Finally, excluded individuals slightly changed the original sample’s age and sex proportions, but differences were subtle.

This research, together with a recently published article [17], by our group, aimed at describing self-perceived health and identifying the main factors related to it. This piece of work contributes to a better understanding on which health, social or contextual variables are relevant for functionally independent older adults.

## Conclusions

This study suggests that several health, social and contextual variables were related to HRQL in a sample of functionally independent older people without cognitive impairment. Specifically, drugs taken daily, sensory-related abilities, leisure activities, social support and housing conditions were the most strongly associated with the outcome of interest. The obtained results support the multidimensional nature of HRQL. Further, it is of special relevance to enhance and advance in the study of factors related to HRQL from a comprehensive point of view. This approach is crucial to the development of health-related programmes for promoting active and healthy ageing and to delay the onset of dependence in this population group.

## Abbreviations

AUC: Area under the curve
BADL: Basic activities of daily living
CI: Confidence intervals
DUFSS: Duke-UNC functional social support questionnaire
EQ-VAS: Visual analogue Scale
GDS: Geriatric depression scale
HRQL: Health-Related quality of life
LSNS: Lubben social network scale
OR: Odds ratio
SD: Standard deviations
STARU: Screening tool for assessing risk of undernutrition
STS: Social trust scale
WHO: World health organization
